# General Internal Assessment of Hand Hygiene Practices Among Diverse Healthcare Personnel in Critical Care and General Wards of a Tertiary Hospital

**DOI:** 10.7759/cureus.93546

**Published:** 2025-09-30

**Authors:** Bilal Masood, Misbah Shirin Memon, Muryyum Dilshad, Tayyaba Rafiq, Muhammad Usairam Cheema, Zahid Azam Chaudry, Kashaf Ali

**Affiliations:** 1 Community Medicine, Bolan University of Medical and Health Sciences, Quetta, PAK; 2 General Medicine, Pakistan Institute of Medical Sciences, Islamabad, PAK; 3 Pathology, Urgent Medical Diagnostics Centre, Rawalpindi, PAK; 4 Oral and Maxillofacial Surgery, Lahore Medical and Dental College, Lahore, PAK; 5 General Medicine, Cheema Surgicare, Gujranwala, PAK; 6 Community Medicine, Nawaz Sharif Medical College, Gujrat, PAK; 7 Pathology, Indus Medical College and Hospital, Tando Muhammad Khan, PAK

**Keywords:** clinical audit, critical care, hand hygiene, infection control, professional practice

## Abstract

Background: Hand hygiene (HH) is a key infection prevention and control (IPC) measure in departments at risk of infection, including pathology and the critical care department. Although the World Health Organization (WHO) has provided its framework of Five Moments of HH, the level of compliance among health workers is uneven. The purpose of this study was to audit HH practice among pathology staff in critical care and general wards through structured observational practice.

Methods: In this cross-sectional observational audit, based on the Five Moments of HH as established by the WHO, the performance of HH compliance was evaluated across various professional groups (medical staff, nursing, and allied health staff). A total of 1,730 HH opportunities were documented among 87 healthcare professionals over the period of six months. Compliance was analyzed by profession, ward type, and shift. While the predictors of compliance were assessed using logistic regression.

Results: Total HH compliance observed was 62.4% (1,080/1,730). Physicians showed the highest compliance (263/348; 75.6%), followed by the allied health professionals (34/49; 69.4%), while nurses (598/1015; 58.9%) and laboratory technicians (185/318; 58.2%) demonstrated lower compliance rates (p<0.001). ICU staff were seen to comply significantly more often than general ward staff (246/346; 71.1% vs. 834/1,384; 60.3%, p<0.001). Compliance was highest during morning shifts (456/692; 65.9%) and lowest at night (246/415; 59.3%, p=0.047). Logistic regression confirmed that professional category, ward type, shift, staffing adequacy, and resource availability were independent predictors of compliance.

Conclusion: The audit revealed moderate HH compliance with significant variation by professional categories and clinical areas. The implementation of behavior-specific interventions and real-time feedback on targeted IPC training is crucial to increase HH adherence and decrease the risk of contracting healthcare-associated infection.

## Introduction

Healthcare-associated infections (HAIs) constitute a long-standing global problem in tertiary care medical settings, resulting in excessive morbidity, prolonged hospital stays (by an average of 8-14 days per patient), and escalated healthcare expenses that generally reach billions of dollars annually worldwide [1]. The World Health Organization (WHO) introduced Five Moments for Hand Hygiene (HH) to develop a structured framework for preventing pathogen transmission during patient care [2]. Although standardized WHO guidelines and hygiene protocols are available, the general compliance among healthcare personnel in most institutions remains below expected levels, with global rates often ranging between 20% and 40% [3]. A low adherence rate has been reported, especially before patient contact (as low as 5-15% in some cases) and in non-critical units, leading to discrepancies in implementation and practice [4].

Several underlying factors that have been established are limited awareness, uneven resource allocation, and the lack of continuous monitoring and feedback [5]. Structured training, behavioral interventions, and provision of alcohol-based hand rubs have shown remarkable improvement in compliance in multiple hospital settings [6]. These advances, however, can prove fleeting in the absence of commitment by organizations or incorporation into routine clinical routines [7]. The advent of technology-aided surveillance (motion sensors, automated counters, and thermal feedback) provides an additional opportunity to measure and enhance HH behaviors objectively [8]. Many audits and interventions target intensive care or surgical areas, although departments that handle large amounts of biological material, like pathology, are underrepresented in these assessments [9]. The hygiene practices of the diagnostic staff must be assessed systematically to ensure safety throughout the continuum of care.

The objective of this study was to determine the HH compliance among the pathology staff based on the WHO Five Moments framework. It assessed compliance within the professional categories and ward types. The study also aimed to identify areas with low-compliance rates to aid in infection control interventions

## Materials and methods

This was a cross-sectional observation audit of HH practice, conducted at the Pathology Department of Indus Medical and Dental College (IMDC) Hospital, Tando Muhammad Khan, Pakistan. The study was approved by the Institutional Review Board of IMDC (Ref: MS/037/04/03/25). The audit process took over six months from November 2024 to April 2025. Two trained infection control officers did the observations as per the standardized framework given by the WHO “Five Moments for Hand Hygiene” [10,11]. Observations were recorded anonymously, and no personal identifiers were noted for any participant at any stage.

The study population included all medical practitioners who worked in the pathology department and were directly involved in patient care. For clarity, professional categories were defined primarily into the following categories: physicians (consultants and residents), nurses (registered nurses, enrolled nurses, auxiliaries, and students), allied health professionals (physiotherapists, psychologists, dietitians, radiographers, and social workers), and laboratory technicians. The departmental workforce register provided a total number of 124 active professionals in the facility by the time of study, including 18 physicians, 42 nurses, 16 allied health professionals, and 48 laboratory technicians.

Out of these categories, 87 individuals were actually observed during the study period in the course of their duties, constituting about 70% of the total department workforce. All categories received a distribution of observations according to the proportion of their patient-care activities. In total, 1,730 HH opportunities were documented, with the highest number of opportunities being expressed by nurses, considering their increased contact with the patients, whilst allied health professionals had fewer opportunities since they are not directly involved in bedside care.

Observers noted the presence or absence of HH at every opportunity (such as before contact with the patient, before an aseptic procedure, after exposure to body fluids, after contact with the patient, after exposure to the patient environment). Where there were several opportunities that occurred in rapid succession, each was noted separately. This operationalization provided inter-observer consistency.

Observations were carried out undeclared in order to reduce the Hawthorne effect, although the possibility of a change in behavior due to awareness of being observed could not be eliminated completely. The systematic time-based sampling strategy was used to provide representativeness in the various shifts. Observation time slots were generated randomly with the help of Microsoft Excel v16.16.22 (Microsoft Corporation, Redmond, Washington, United States), and observation sessions were held during the morning, evening, and night shifts to identify potential variations in practice. General wards as well as intensive care units (ICUs) were included. In situations where there was a difference in the number of observations between settings, there was an indication of the natural disparity of patient turnover and staff availability during observation.

The WHO definition of HH opportunities was applied to each observation. For each opportunity, the observers documented whether HH was conducted (by washing hand with soap and water or by applying alcohol-based hand rub), the category of the professional health workers on the ward (general ward or ICU), the shift (morning, evening, or night), the staff to patient ratio at the time, and the presence of resources like sinks or alcohol dispensers within easy access.

A structured proforma was used for documentation of all observations, which were then entered into IBM SPSS Statistics for Windows, version 26.0 (Released 2019; IBM Corp., Armonk, New York, United States) for analysis. Both descriptive and inferential analyses were conducted. Descriptive statistics were expressed as frequencies and percentages of compliance rates by the professional category, type of ward, and shift. Group differences were examined using inferential statistics. Comparative compliance across the professional categories, types of wards, and shifts was done using chi-square tests. Binary logistic regression was performed to investigate predictors of compliance, whereby instances of professional category, type of ward, shift, staffing ratio, and availability of resources were taken as independent variables. A p-value of less than 0.05 was regarded as significant.

## Results

A total of 1,730 HH opportunities were recorded among the 87 healthcare professionals over a period of six months. Overall compliance was seen as 62.4% (n=1,080). Table 1 provides the distribution of professionals observed and their total HH opportunities.

**Table 1:** Distribution of healthcare professionals and hand hygiene opportunities HH: hand hygiene

Professional Category	Number of Staff Observed (n=87), n (%)	HH Opportunities (n=1730), n (%)
Physicians	15 (17.2%)	348 (20.1%)
Nurses	50 (57.5%)	1,015 (58.6%)
Allied Health Professionals	7 (8.0%)	49 (2.8%)
Laboratory Technicians	15 (17.2%)	318 (18.4%)

The findings showed that nurses had the majority of HH opportunities (n=1,015; 58.6%), followed by physicians (n=348; 20.1%), laboratory technicians (n=318; 18.4%), and allied health professionals (n=49; 2.8%). Table 2 presents HH compliance by professional group.

**Table 2:** Hand hygiene compliance by professional category χ² = Chi-square test; **p<0.001 highly significant, Chi-square is designed to test associations across categories in a contingency table, not within a single row. HH: hand hygiene

Professional Category	HH Opportunities, n	HH Performed, n	Compliance Rate (%)	χ² / Test Value	p-value
Physicians	348	263	75.6	-	-
Nurses	1,015	598	58.9	-	-
Allied Health Professionals	49	34	69.4	-	-
Laboratory Technicians	318	185	58.2	-	-
Total	1,730	1,080	62.4	28.47	<0.001**

The highest compliance was seen in physicians (263/348; 75.6%), which was followed by allied health professionals (34/49; 69.4%). Nurses and laboratory technicians demonstrated relatively lower compliance at 598/1,015 (58.9%) and 185/318 (58.2%), respectively. The test values confirmed a statistically significant difference in compliance across professional categories (χ² = 28.47, df = 3, p < 0.001). Table 3 outlines compliance in general wards and ICUs.

**Table 3:** Hand hygiene compliance by ward type χ² = Chi-square test; **p<0.001 highly significant, Chi-square is designed to test associations across categories in a contingency table, not within a single row. ICU: intensive care unit; HH: hand hygiene

Ward Type	HH Opportunities (n)	HH Performed (n)	Compliance Rate (%)	χ² Value	p-value
General Ward	1,384	834	60.3	–	–
ICU	346	246	71.1	–	–
Total	1,730	1,080	62.4	12.89	<0.001**

ICU staff demonstrated significantly higher compliance (246/346; 71.1%) compared to general ward staff (834/1,384; 60.3%). This difference was also statistically significant according to the chi-square test (χ² = 12.89, df = 1, p < 0.001). Table 4 presents compliance across shifts.

**Table 4:** Hand hygiene compliance by shift χ² = Chi-square test; n = size;  p<0.05 significant, Chi-square is designed to test associations across categories in a contingency table, not within a single row. HH: hand hygiene

Shift	HH Opportunities (n)	HH Performed (n)	Compliance Rate (%)	χ² Value	p-value
Morning	692	456	65.9	–	–
Evening	623	378	60.7	–	–
Night	415	246	59.3	–	–
Total	1,730	1,080	62.4	6.12	0.047*

In the morning shift, the compliance was seen highest (456/692; 65.9%), followed by evening (378/623; 60.7%), and lowest during night shifts (246/415; 59.3%). The difference was statistically significant (χ² = 6.12, df = 2, p = 0.047). Table 5 demonstrates compliance with the WHO Five Moments.

**Table 5:** Compliance with WHO Five Hands Moment HH: hand hygiene Reference: WHO, 2021 [11]

WHO Five Moments	HH Opportunities (n)	HH Performed (n)	Compliance Rate (%)
Before patient contact	420	230	54.8%
Before an aseptic task	310	190	61.3%
After body fluid exposure risk	210	150	71.4%
After patient contact	400	260	65.0%
After contact with patient's surroundings	390	250	64.1%
Total	1,730	1,080	62.4%

Moment-specific analysis revealed that the lowest compliance was before patient contact (230/420; 54.8%), and the highest was after body fluid exposure (150/210; 71.4%), as well as after contact with patient surroundings (250/390; 64.1%). A binary logistic regression model was fitted to identify independent predictors of HH compliance (as shown in Table 6).

**Table 6:** Binary logistic regression predictors of HH compliance *p<0.05  significant; **p<0.001 highly significant; Logistic regression model χ² = 156.23, df = 9, p < 0.001. ref: reference category; AOR: adjusted odds ratio; ICU: intensive care unit; HH: hand hygiene

Variable	Category	Adjusted Odds Ratio (AOR)	95% CI	p-value
Professional Category	Physicians (ref)	1.00	–	–
	Nurses	0.476	0.344–0.659	<0.001**
	Allied Health	0.742	0.347–1.588	0.441
	Laboratory Technicians	0.439	0.310–0.622	<0.001**
Ward Type	ICU vs General Ward	1.669	1.284–2.171	<0.001**
Shift	Morning (ref)	1.00	–	–
	Evening	0.802	0.630–1.021	0.072
	Night	0.750	0.571–0.986	0.039*
Staffing Ratio	Adequate vs Stretched	1.489	1.204–1.842	<0.001**
Resource Availability	Available vs Not	1.687	1.355–2.100	<0.001**

In comparison to physicians, nurses (AOR 0.476, p<0.001) and laboratory technicians (AOR 0.439, p<0.001) were seen as significantly less likely to comply. On the other hand, ICU staff were 1.67 times more likely to comply in comparison to general ward staff. Night shift workers had lower odds of compliance as compared to the morning shift (AOR 0.750, p=0.039). Adequate staffing (AOR 1.489, p<0.001) and resource availability (AOR 1.687, p<0.001) were seen as strong positive predictors.

## Discussion

This study aimed to explore compliance with HH among pathology personnel as defined by the WHO under the Five Moments and to determine the compliance variations by professional categories, ward type, and clinical moments. The findings suggested that HH practices were moderately implemented, with trends indicating variations across different wards and professions.

The results of the moment-specific analysis showed that HH observance was lowest before interacting with the patient and highest after contact with the environment of the patient. These findings are demonstrated by studies showing that medical professionals tend to underestimate the significance of pre-contact HH and fail to recognize risk factors associated with invisible contamination. Another study showed that, rather than preventative, HH practices tend to be reactive and are influenced by local context rather than standard operating procedures [12]. Among all workers, medical staff compliance rates were higher than those of nursing and allied health workers. This trend correlates with previous studies defining medical professionals as having greater infection prevention and control-related training opportunities and being more often placed under audit [13]. Nevertheless, this difference may also be related to hierarchical norms and perceived responsibilities related to physician roles [14]. Other subgroups, including pediatricians and radiographers, had high compliance percentages, but due to the very small sample size, it should not be overinterpreted, though these trends aligned with current studies [15].

Across wards, the ICU ward appeared to have higher compliance than general wards, contrary to other studies where ICU staff achieved higher compliance due to stringent protocols and monitoring [16]. This inconsistency can be explained by a higher cognitive burden in critical contexts, which causes protocols to be neglected [17]. Other research results indicate that visible HH tools, such as dispensers and posters, achieve higher compliance levels when used with behavioral interventions [18]. These results suggest that individual awareness and contextual support are factors that can encourage HH adherence [19]. Effective techniques should consider a combination of educational measures, leadership roles, and behavior reinforcement [20]. Upon introducing real-time monitoring and feedback, it has demonstrated success in improvement [21].

This audit had a number of limitations: some subgroups were too small to make comparisons; the single-center design and direct observation could minimize external validity and bias of the findings of the research. Unmeasured contextual factors and the potential presence of the Hawthorne effect meant that the results were to be understood as indicative, but not conclusive. Other confounding variables, including workload and environmental factors, were not measured. Future studies are recommended to employ longitudinal and multi-center designs to determine the sustainability of interventions and understand the effect of monitoring of hygiene on patient safety and infection risks.

## Conclusions

HH is an essential element of infection prevention, but this study documented an overall compliance rate of only 62.4% among pathology staff. Adherence was low before patient contact, and high after touching patient surroundings, with medical staff showing a higher rate of compliance than nursing and allied health personnel. General wards performed better compared to critical care units, indicating discrepancies in high-risk settings. The findings emphasize that specific training, behavioral interventions, and real-time feedback systems are much needed to enhance compliance.
